# High Treg development in the first year of life gives insight into immune regulation

**DOI:** 10.1186/1546-0096-12-S1-P43

**Published:** 2014-09-17

**Authors:** Sytze De Roock, Krijn Dijkstra, Sanne Hoeks, Berent Prakken

**Affiliations:** 1 Laboratory of Translational Immunology, UMC UTRECHT, Utrecht, Netherlands

## Introduction

Juvenile Idiopathic Arthritis (JIA) pathology is characterized by a disregulated adaptive immune system. This fact is exemplified by reduced regulatory T cell (Treg) functionality and increased T helper (Th)17 cell activity.

## Objectives

In order to further understand the relation between Treg and Th17 development, we investigated the induction of these cells from naïve T cells in early human life.

## Methods

For this, we compared cells from umbilical cord blood (CB) with cells from adult volunteers, as well as cells from schisis patients who undergo several corrective operations in early life. We cultured these cells with antiCD3 for 5 days and subsequently investigated T cell subtype induction by flow cytometry.

## Results

We show that upon activation, CB cells easily adopt a Treg phenotype, whereas Th17 cells can not be induced. Although production of inflammatory cytokines is dramatically lower in CB cells, addition of Th17 inducing cytokines IL-1b or IL-6 did not influence this phenomenon. Instead, an increased programmed death (PD)-1 signaling and decreased Th17 defining transcription factor RORC activation underlie the prevention of inflammatory T cell activity. The propensity for Treg development is a phenomenon which we still observed in samples from 12 month old children, whereas we found Th17 cell development as early as three months after birth. This development in the first year of life is likely related to prevention of aberrant T cell responses towards self and the developing microbiome.

## Conclusion

These data give more insight into the development of the immune system and inflammation and can lead to novel targets in JIA therapy, aiming at the balance between Treg and Th17 cells.

## Disclosure of interest

None declared.

